# The relevance of ‘mixed anxiety and depression’ as a diagnostic category in clinical practice

**DOI:** 10.1007/s00406-016-0684-7

**Published:** 2016-03-22

**Authors:** Hans-Jürgen Möller, Borwin Bandelow, Hans-Peter Volz, Utako Birgit Barnikol, Erich Seifritz, Siegfried Kasper

**Affiliations:** 1 Clinic and Polyclinic for Psychiatry and Psychotherapy, Ludwig Maximilian University, Nußbaumstrasse 7, 80336 Munich, Germany; 2 Department of Psychiatry and Psychotherapy, University Medical Center Göttingen, von-Siebold-Strasse 5, 37075 Göttingen, Germany; 3 Hospital for Psychiatry, Psychotherapy and Psychosomatic Medicine Schloss Werneck, Balthasar-Neumann-Platz 1, 97440 Werneck, Germany; 4 Research Unit Ethics, Institute for the History of Medicine and Medical Ethics, University Medical Center Cologne, Cologne, Germany; 5 Department of Child and Adolescent Psychiatry, Psychosomatic and Psychotherapy, Albertus Magnus University of Cologne, Joseph Stelzmann Strasse 20, 50937 Cologne, Germany; 6 Department of Psychiatry, Psychotherapy, and Psychosomatics, Psychiatric Hospital, Lenggstrasse 31, 8032 Zurich, Switzerland; 7 Department of Psychiatry and Psychotherapy, Medical University of Vienna, Währinger Gürtel 18-20, 1090 Vienna, Austria

**Keywords:** Mixed anxiety and depression, Subthreshold anxiety, Subthreshold depression, Classification, Psychiatric disorder

## Abstract

According to ICD-10 criteria, mixed anxiety and depressive disorder (MADD) is characterized by co-occurring, subsyndromal symptoms of anxiety and depression, severe enough to justify a psychiatric diagnosis, but neither of which are clearly predominant. MADD appears to be very common, particularly in primary care, although prevalence estimates vary, often depending on the diagnostic criteria applied. It has been associated with similarly pronounced distress, impairment of daily living skills, and reduced health-related quality of life as fully syndromal depression and anxiety. Although about half of the patients affected remit within a year, non-remitting patients are at a high risk of transition to a fully syndromal psychiatric disorder. The validity and clinical usefulness of MADD as a diagnostic category are under debate. It has not been included in the recently released DSM-5 since the proposed diagnostic criteria turned out to be not sufficiently reliable. Moreover, reviewers have disputed the justification of MADD based on divergent results regarding its prevalence and course, diagnostic stability over time, and nosological inconsistencies between subthreshold and threshold presentations of anxiety and depressive disorders. We review the evidence in favor and against MADD and argue that it should be included into classification systems as a diagnostic category because it may enable patients to gain access to appropriate treatment early. This may help to reduce patients’ distress, prevent exacerbation to a more serious psychiatric disorder, and ultimately reduce the societal costs of this very common condition.

## Background

It has been estimated that about 85 % of patients with depression also experience significant symptoms of anxiety. Similarly, symptoms of depression occur in up to 90 % of patients with anxiety [1]. Co-morbid anxiety and depression may occur at any age, from childhood and adolescence [2] to old age [3]. They are more disabling, more resistant to treatment, have a greater risk of suicide, and are associated with more severe psychological, physical, social, and workplace impairment than either condition alone [e.g., 4].

By ‘depression’ and ‘anxiety,’ we refer to clinically relevant disorders whose symptoms may cause profound suffering and may interfere with essential activities of daily living. A clinical picture characterized by a combination of symptoms of anxiety and depression severe enough to justify a psychiatric diagnosis, neither of which are clearly predominant and which, when considered separately, do not meet the full diagnostic criteria of either syndromal anxiety (e.g., generalized anxiety disorder, GAD) or depressive disorder (e.g., major depressive disorder, MDD), may be diagnosed as mixed anxiety and depressive disorder (MADD) according to the criteria of the currently valid 10th revision of the International Statistical Classification of Diseases and Related Health Problems [ICD-10; 5].

Patients meeting the diagnostic criteria of MADD are particularly common in primary care [6, 7]. Although there is some variation in the estimated prevalence rates, researchers widely agree that MADD is among the most prevalent psychiatric disorders [e.g., 8, 9].

As will be argued below, patients suffering from subsyndromal psychiatric conditions, including MADD, have been shown to suffer from similarly pronounced distress, impairment of daily living skills, and reduced health-related quality of life as individuals with a fully syndromal disorder [e.g., 7, 10]. Moreover, co-occurring depression and anxiety are associated with higher disability scores and co-morbid physical conditions than having anxiety or depression alone [e.g., 11].

Despite the undisputedly high prevalence of co-occurring, subsyndromal symptoms of anxiety and depression in the community, the question of whether MADD is a scientifically valid, clinically useful, and justified diagnosis in its own right is under scrutiny, and there is an ongoing debate as to whether it deserves a place in psychiatric nosology. In this article we make a case for retaining MADD as an independent and valid diagnosis and present arguments why it should (continue to) be included into psychiatric classification systems.

## MADD in history

From the beginning of psychiatric nosology in the late nineteenth century until the 1970s, anxiety and depression were widely accepted in the non-psychoanalytic psychiatric community as different manifestations of one affective spectrum disorder [12]. As a matter of fact, German psychiatrist Emil Kraepelin, whose work in the late nineteenth century is recognized as seminal for modern classification systems of mental diseases, perceived anxiety to be a fundamental part of all psychiatric illnesses and therefore did not consider it to be an independent disorder [13]. The rise of psychopharmacology that started in the 1950s led to the development of drugs that had a relatively specific antidepressant (e.g., tricyclics) or anxiolytic effect (e.g., benzodiazepines), and supported a dichotomization between depression and anxiety [12]. During the development of the 3rd edition of the Diagnostic and Statistical Manual of Mental Disorders [DSM-III; [14]], the drafting of the sections on depressive and anxiety disorders was assigned to 2 different advisory committees, which contributed to the fact that anxiety and depression were included as completely different disorders in DSM-III, first released in 1980 [15].

In 1990 the World Health Organization (WHO) endorsed ICD-10 [5], which, unlike its predecessor ICD-9, accepted in 1978, included MADD in its section on anxiety disorders as a diagnostic category (F41.2; Table 1). The original definition was later complemented by a set of diagnostic guidelines for primary care [16] where patients with MADD are particularly common (Table 2).

**Table 1:** Mixed anxiety and depressive disorder in ICD-10 [5]

F41.2—Mixed anxiety and depressive disorder
*Definition*
This category should be used when symptoms of anxiety and depression are both present, but neither is clearly predominant, and neither type of symptom is present to the extent that justifies a diagnosis if considered separately. When both anxiety and depressive symptoms are present and severe enough to justify individual diagnoses, both diagnoses should be recorded and this category should not be used
*Inclusion*
Anxiety depression (mild or not persistent)

**Table 2:** Mixed anxiety and depressive disorder—diagnostic guidelines for ICD-10 in primary care [16]

*Presenting complaints*
The patient presents with variety of symptoms of anxiety and depression
There may initially be one or more physical symptoms (e.g., fatigue, pain). Further enquiry will reveal depressed mood and/or anxiety
*Diagnostic features*
Low or sad mood
Loss of interest or pleasure
Prominent anxiety or worry
The following associated symptoms are frequently present: disturbed sleep, tremor, fatigue or loss of energy, palpitations, poor concentration, dizziness, disturbed appetite, suicidal thoughts or acts, dry mouth, loss of libido, tension, and restlessness
*Differential diagnosis*
If more severe symptoms of depression or anxiety are present, see management guidelines for Depression—F32 and Generalized anxiety—F41.1
If somatic symptoms predominate, see Unexplained somatic symptoms—F45
If the patient has a history of manic episodes (excitement, elevated mood, rapid speech), see Bipolar disorder—F31
If heavy alcohol or drug use is present, see Alcohol use disorders—F10 and Drug use disorders—F11

Due to the large number of reports in the literature, and possibly also as a reaction to ICD-10, the DSM-IV Anxiety Disorders Workgroup proposed MADD for inclusion into DSM-IV [17]. A subsequently initiated field trial showed that MADD is seen frequently in clinical practice and involves significant distress, impairment, and increased risk of evolution to a more serious condition [18]. However, the DSM-IV task force decided to place the diagnosis in the research appendix, due to lack of information about its concurrent or predictive validity, interrater reliability, and prevalence in the general population [17], when the manual was finally published in 1994 [19].

The DSM-IV diagnostic research criteria for MADD are shown in Table 3. When DSM-5 was being developed, the Mood Disorders Workgroup again proposed to include MADD as an ‘official’ diagnostic category [17]. The criteria proposed by the workgroup (Table 4) were, however, distinctly different from both those of the DSM-IV research appendix and the ICD-10 criteria, with the disadvantage that, for obvious reasons, there were no empirical data from studies in which these newly proposed criteria had ever been applied. The new criteria were evaluated in the context of the DSM-5 field trials, where the diagnosis of MADD was assessed to be not sufficiently reliable [20, 21], which probably did not come as much of a surprise given the fact that there was practically no clinical experience in the application of the newly proposed criteria. Therefore, MADD was not included into the final version of DSM-5 released in 2013 [22].

**Table 3:** DSM-IV research criteria for mixed anxiety depressive disorder [19]

Persistent or recurrent dysphoric mood for at least 1 month
The dysphoric mood is accompanied by at least 1 month of four (or more) of the following symptoms
Difficulty concentrating or mind going blank
Sleep disturbance (difficulty falling asleep or staying asleep, or restless unsatisfying sleep)
Fatigue or low energy
Irritability
Worry
Being easily moved to tears
Hypervigilance
Anticipating the worst
Hopelessness
Low self-esteem or feelings of worthlessness
The symptoms cause clinically significant distress or impairment in social, occupational, or other important areas of functioning
The symptoms are not a result of the direct physiological effects of a substance or a general medical condition
All of the following
Criteria have never been met for major depressive disorder, dysthymic disorder, panic disorder, or GAD
Criteria are not currently met for any other anxiety or mood disorder (including an anxiety or mood disorder in partial remission)
The symptoms are not better accounted for by any other mental disorder

**Table 4:** Proposed criteria for DSM-5 mixed anxiety depression [from 17, Appendix II]

(1) The patient has three or four of the following symptoms for at least 2 weeks, one of which must be (a) or (b)
(a) Depressed mood most of the day, almost every day, as indicated by either subjective report (e.g., feels sad or empty) or observation made by others (e.g., appears tearful)
(b) Markedly diminished interest or pleasure in all, or almost all, activities most of the day, almost every day
(c) Significant weight loss when not dieting or weight gain (e.g., a change of more than 5 % of body weight in 1 month), or decrease or increase in appetite almost every day
(d) Insomnia or hypersomnia almost every day
(e) Psychomotor agitation or retardation nearly every day (observable by others, not merely subjective feelings of restlessness or being slowed down)
(f) Fatigue or loss of energy nearly every day
(g) Feelings of worthlessness or excessive or inappropriate guilt (which may be delusional) nearly every day (not merely self-reproach or guilt about being sick)
(h) Diminished ability to think or concentrate, or indecisiveness, almost every day (either by subjective account or as observed by others)
(i) Recurrent thoughts of death (not just fear of dying), recurrent suicidal ideation without a specific plan, or a suicide attempt or a specific plan for committing suicide
(2) The symptoms in (1) are accompanied by two (or more) of the following symptoms of anxious distress, also lasting at least 2 weeks:
(a) Irrational worry
(b) Preoccupation with unpleasant worries
(c) Having trouble relaxing
(d) Motor tension
(e) Fear that something awful would happen
(3) No other DSM diagnosis of anxiety or depression is present

A survey performed jointly by the World Psychiatric Association (WPA) and the WHO among nearly 5000 psychiatrists in 44 countries showed that MADD was the 4th most frequently used diagnostic category, but was among those with the lowest goodness of fit or accuracy and among the most difficult to use [23]. In view of the currently still ongoing revision of ICD it has therefore been suggested to maintain the category of MADD but to provide more explicit guidance for its diagnosis [24, 25].

According to the current draft version of ICD-11 [26], whose final version has now been scheduled for release in 2017, the classification system will continue to include a diagnostic category for subsyndromal, co-morbid anxiety, and depression, which will, however, be moved from the anxiety disorders to the depressive disorders section and, accordingly, will be renamed ‘mixed depressive and anxiety disorder.’ Moreover, in response to previous criticism that the definition of MADD in ICD-10 was too vague [e.g., 7, 24, 27], a more elaborate set of criteria is being proposed (Table 5).

**Table 5:** Proposed criteria for ICD-11 mixed depressive and anxiety disorder as of August 6, 2015 [26, foundation ID : http://id.who.int/icd/entity/314468192 ]

7A73 Mixed depressive and anxiety disorder
*Definition*
Mixed depressive and anxiety disorder is characterized by symptoms of both anxiety and depression more days than not for a period of 2 weeks or more. Neither set of symptoms, considered separately, is sufficiently severe, numerous, or persistent to justify a diagnosis of a depressive episode, dysthymia, or an anxiety and fear-related disorder. Depressed mood or diminished interest in activities must be present accompanied by additional depressive symptoms as well as multiple symptoms of anxiety. The symptoms result in significant distress or significant impairment in personal, family, social, educational, occupational, or other important areas of functioning. There have never been any prior manic, hypomanic, or mixed episodes, which would indicate the presence of a bipolar disorder
*Inclusion*
Anxiety depression (mild or not persistent)

## Anxiety and depression: two sides of the same coin?

There is both neurobiological and phenomenological evidence that depression and anxiety may represent different manifestations of a similar vulnerability that has been linked to a general ‘distress’ factor [7, 11, 28–30]. Already in 1991, Clark and Watson [31] proposed a tripartite model of affective disorders consisting of a general distress factor, physiological hyperarousal (specific to anxiety), and anhedonia (specific to depression).

In clinical as well as epidemiological studies, anxiety and depression have consistently shown considerable symptom overlap [e.g., 32, 33]. Moreover, it has been observed that in a longitudinal perspective diagnostic conversion between anxiety and depressive disorders is not unlikely to occur [34, 35]. It has therefore been suggested that the two conditions may be regarded as the extremes of one continuum [30] with a shared diathesis best described as non-specific ‘negative affect’ [36, 37].

The presence of psychiatric symptoms has been found to coincide with specific neurochemical variations that are independent of the clinical diagnosis [38–40]. The approach resembles the concept of target symptoms described by Freyhan already in 1979, according to which psychotropic drugs act on symptoms rather than on disorders [41] and may therefore be efficacious in different disorders that share common symptoms. The interpretation is supported by the observation that newer-generation antidepressant drugs have been demonstrated to be efficacious in anxiety disorders as well. This is particularly true for selective serotonin reuptake inhibitors (SSRIs) whose efficacy in anxiety and depression has been linked to their agonistic action on the serotonin-1A receptor subtype (5-HT1A) [42–44], and the difference between their antidepressant and anxiolytic effect may depend on whether they act on pre-synaptic (anxiolytic) or post-synaptic (antidepressant) 5-HT1A receptors [45]. Consequently, SSRIs, that were originally developed as antidepressants [46], are now also recommended as first-line treatment for anxiety disorders [e.g., 47], and there is also evidence that SSRIs are efficacious in MADD where studies have been performed for sertraline [48], fluvoxamine [49], and citalopram [50]. Comparable results were also found for Silexan, a herbal active substance acting on the serotonin-1A receptor subtype [51], which was shown to be efficacious in anxiety disorders [52, 53] as well as in MADD [54].

Interestingly, investigations including cortical areas with high expression of serotonergic receptor subtypes provide further evidence for the argumentation that two extremes of one continuum are involved in the MADD mode of neuronal activations and inhibition in the CNS [55, 56]. In addition, the orbito-frontal cortex (OFC) has a dense serotonin innervation, and it is widely accepted that the inhibitory control functions of the OFC are disrupted by serotonin. A novel mode of investigating cortical dynamics [57], in correlation with transmitter receptor fingerprints [58] in GAD, depression and MADD, respectively, may help to develop more specific and effective strategies by accomplishing pharmacological studies for treating both sides of one coin: anxiety and depression, focused on the coin itself: MADD.

The hypothesis of two extremes switching in one continuum, which is expressed in MADD, could be strengthened by neuroscientific findings of neuronal over-arousal in the anxious states and neuronal under-arousal in the depressive states, including impairment of intentional control of neural functioning [55] and self-focus in two segregated cortical subareas of one functionally distinct area, the OFC [56, 57]. Sensory cue inputs in depression and anxiety-related internal brain activities govern the firing of OFC neurons. Furthermore, the theta activity in the OFC is related to self-referenced processing in depression and to aversive processing in anxiety, reflecting two extremes in one neural continuum [57].

Independently of these findings, the genetic matching theory of MADD by Kendler and colleagues [59] provides evidence for the same genetic origin of anxiety and depression by shared genetic factors expressed in vulnerable patients (see [60], p. 13).

Clinical studies concerning the systematic analysis of both shared and separate specific symptoms based on genetic matchings [28, 59, 61] and on receptor fingerprints [58] in investigating anxiety and depression may help resolve the current controversial debate. This would foster the inclusion of MADD as an accepted epidemiological hypothesis in pharmacological treatment studies with specific designs in GAD, depression, and the combination of both (MADD), as a scientifically and clinically valid common entity [60–62].

Classification systems may thus benefit from an extension of the current, mainly phenomenology-based approach by the incorporation of reliably neurobiological findings, taking onto account the interrelationship between psychological, social, and cultural factors on the one hand and biochemistry and physiology findings on the other.

## Clinical evidence

Studies investigating the validity of the diagnosis of MADD according to the criteria of ICD-10 and the DSM-IV research appendix have produced partly conflicting results.

Of 796 consecutive primary care clinic attendants without known psychiatric illness interviewed by Stein and colleagues [63], 78 were further investigated systematically and 10 (12.8 %) met the ICD-10 or the authors’ own criteria for MADD.

For the UK it has been estimated that co-occurring, subthreshold symptoms of anxiety and depression make up almost half of all psychological problems and are 4 times more common than depression alone [64].

In a longitudinal, naturalistic study of anxiety disorders in primary care reported by Weisberg et al. [65], patients screened positive for anxiety symptoms were also interviewed for MADD, and only 4 out of 1634 participants (0.2 %) met the DSM-IV research criteria, with an adjusted probability of remission at 1 year after diagnosis of 80 %.

Using data of 1183 patients with various anxiety and depressive disorders from the WHO Collaborative Study on psychological problems in general health care [66], Barkow et al. [27] identified 85 cases with MADD according to ICD-10 criteria and found that only 1 of them still met the diagnostic criteria for MADD after 1 year. Whereas about half of the remaining patients remitted, about one quarter developed a syndromal anxiety and/or depressive disorder, while the remaining patients were diagnosed as having some other ICD-10 psychiatric disorder. The authors concluded that patients suffering from MADD are either only transiently affected or in a prodromal or residual state of a syndromal affective disorder. It should be noted, however, that the 1-year recovery rates reported by Barkow et al. [27] for patients with syndromal depressive or anxiety disorders were comparable to those of patients with MADD although transition to another ICD-10 disorder was lower.

Similar observations were also published by Spijker et al. [67], based on health survey data from the Netherlands, who estimated a prevalence of MADD (defined on the basis of the DSM-IV research criteria) of 0.6 % in the general population and concluded that MADD is not a relevant diagnosis in terms of prevalence and consequences when classified according to the currently proposed criteria which exclude patients with previous relevant psychopathology. By contrast, Usall and Marquez [68], using the DSM-IV research criteria, found MADD to be comparatively stable during 12-month follow-up.

Using taxometric methods, Schmidt et al. [69] analyzed data from a school-based sample of 706 adolescents in which they found a prevalence of MADD (according to the DSM-IV research criteria) around 13 %. Their results also support previous findings according to which MADD may be predictive of subsequent syndromal anxiety and depressive disorders [e.g., 70].

In a survey of more than 21,000 patients who attended primary care for any reason, Balestrieri et al. [71] identified 1.8 % of subjects with MADD, more than half of whom had no history of any anxiety or depression disorder. The results dispute the hypothesis that co-morbid, subsyndromal anxiety and depression should be viewed as a state of partial remission of a previous syndromal disorder.

Based on representative population survey data of 8580 respondents in Great Britain, Das Munshi et al. [7] found MADD (defined in accordance with ICD-10) to account for about half of the cases of common mental disorder, with a 1-month prevalence of 8.8 % and an impact on health-related quality of life comparable to that of syndromal anxiety or depression. Using latent class analysis, the authors found considerable overlap between patients with MADD, those with depression and co-morbid anxiety, and those with anxiety and co-morbid depression, based on the presence of negative affect. The authors interpreted their results as challenging the notion of these conditions as having distinct phenomenologies.

Although Hettema et al. [72] fully implemented the DSM-IV and ICD-10 requirement that MADD could not be diagnosed if the subject had met prior lifetime criteria for a full mood or anxiety disorder, their latent class analyses of data from more than 7500 participants of the population-based Virginia Adult Twin Study of Psychiatric and Substance-Use Disorders [73] detected MADD prevalence rates of around 10 %. Comparing classification results of the 2 ‘waves’ of the survey performed about 17 months apart, they found that 47 % of the subjects classified as having MADD during the first wave were in remission during the second, 23 % had persistent MADD, and 30 % had developed syndromal anxiety or depressive disorder. Moreover, MADD was found to be significantly associated with childhood adversity, poor parenting, lifetime traumas, recent life events, high neuroticism, co-morbid substance-use disorders, and familial aggregation. The authors conclude that MADD is a commonly occurring, identifiable, syndromal subtype that should be considered for inclusion in future nosological systems.

Similar rates of remission or progression in patients with ICD-10 compliant MADD were observed as well by Walters et al. [74] who also found that patients with MADD were twice as likely to have significant distress and persistently lower mental health-related quality of life at 3-month follow-up than participants with no psychiatric diagnosis.

In a study performed in Germany, data from a random sample comprising 13.2 % of all 10,162,162 patients registered in the Ambulatory Health Care System in 2008 were analyzed. Of all psychiatric diagnoses made by mental health specialists, ICD-10 MADD was the most commonly diagnosed anxiety disorder (7.2 %), followed by panic disorder (4.4 %) and GAD (2.5 %) [75, 76]. However, the quality of these diagnoses was not scrutinized, so that it cannot be excluded that in many cases MADD was diagnosed although the patients fulfilled the full criteria for both syndromal depression and an anxiety disorder.

While the fact that a substantial number of individuals in the community, and notably of those seeking primary care, suffer from co-morbid symptoms of anxiety and depression is undisputed, the results regarding the prevalence of anxiety/depression co-morbidity meeting the criteria of MADD are discrepant and partly confusing. Since ICD-10 and DSM-IV research criteria for MADD differ substantially, it is not surprising that studies based on one set of criteria find prevalence rates and phenomenology different from those using the other set. Moreover, some studies have excluded patients with a lifetime history of other anxiety or depressive disorders [67, 72], while others have not [e.g., 7, 69]. Nevertheless, there is also heterogeneity between some studies using the criteria from the same classification system.

Preskorn and Fast [77] have identified 2 main factors contributing to the difficulty to distinguish reliably between anxiety and depressive disorders, (a) the fact that diagnoses are mainly based on cross-sectional assessments and that the cross-sectional criteria for both disorders overlap considerably, and (b) lack of focus on the longitudinal course of the patients’ complaints to which, according to Preskorn and Fast [77], particularly primary care physicians do not devote enough time and are not adequately trained. While the authors conclude that a diagnostic category of MADD is not helpful and may even encourage cursory evaluation of patients rather than motivate physicians to reliably distinguish between anxiety and depression, their findings may also be interpreted in favor of a better operationalization of the diagnostic criteria of MADD, particularly for use in primary care.

It should also be noted that the true prevalence of MADD is likely underestimated by community-based studies since affected individuals frequently present in primary care with somatic, rather than psychological, complaints [78].

## Nosological issues

There is an ongoing debate as to whether a diagnostic category of MADD is required for appropriately classifying patients with co-morbid, subthreshold anxiety and depression. While some researchers prefer a simultaneous classification of subthreshold anxiety disorder and subthreshold depression [e.g., 67, 79], others advocate a choice between the two [e.g., 77].

The proposal to code subthreshold anxiety and subthreshold depression separately implies the notion that the patient is suffering from 2 independent, readily distinguishable, and thus separately codable disorders. It should be noted, though, that throughout the history of psychiatric classification neurotic depression and anxiety were perceived to be closely related concepts [13, 15] that were placed in 2 different classes of disorders only in DSM-III. As shown above, both phenomenological and neurobiological evidence suggest that such a strict separation may not be appropriate as anxiety and depression may be better explained as the extremes of a continuum characterized by non-specific distress or negative affect. Moreover, it is worth mentioning that current classification systems do not even include dedicated diagnostic categories for either subthreshold depression or anxiety. Instead, these presentations have to be coded in a residual, ‘catch all’ category like ‘Depressive/anxiety disorder, unspecified’ (ICD-10 F32.9 and F41.9) or ‘Other specified depressive/anxiety disorder’ (DSM-5), the combination of which is clearly not an appropriate, unequivocal characterization of the phenomenology observed in MADD.

As regards the second proposal, to classify patients with co-morbid, subthreshold depression and anxiety as either suffering from anxiety or depressive disorder, epidemiological studies indicate that approximately half of the patients with MADD develop a syndromal psychiatric disorder within about a year [27, 72]. Therefore, it has been argued that if MADD is actually a prodromal stage of another psychiatric disorder, it should be classified in accordance with this disorder, not as an independent diagnostic concept [79]. While this argument is perfectly understandable from a research perspective, the question of whether a particular patient presenting with symptoms meeting the criteria of MADD will progress to some syndromal disorder, and to which disorder she/he is likely to progress, requires a longitudinal perspective that cannot always be obtained from the patient’s history and would rather necessitate a look into the future. In clinical practice, a ‘forced choice’ between anxiety (with co-morbid depression) and depression (with co-morbid anxiety) will likely lead to arbitrary decisions when both components are equally important and only cross-sectional and history information is available.

Batelaan et al. [79] have noted that there is a certain inconsistency in defining MADD as a diagnostic category for co-morbid, subthreshold anxiety and depression, while, at the same time, there is no comparable option when both components are equally important at the threshold level (e.g., a clinical presentation meeting the criteria of both MDD and GAD). Indeed, Tyler [29] has already pointed out that the discussion about the usefulness of MADD as a diagnostic category has mainly be focused on coexisting, subsyndromal presentations. He suggested that there should also be a diagnostic category for coexisting, syndromal GAD and MDD for which he proposed the term ‘cothymia.’ Moreover, Hettema and colleagues have observed that symptom endorsement patterns differed between threshold and subthreshold anxiety and depressive disorders primarily in a quantitative rather than qualitative manner, suggesting MADD symptomatology may be better represented as a dimensional continuum [72]. Along the same line, a double threshold concept has been suggested which defines one threshold for mental disorder and a second (lower) threshold for mental health [80]. The resulting between-threshold zone of subthreshold mental illness could be useful in applying treatment according to disease severity, optimizing cost-effectiveness, and controlling the burden to the healthcare system [81].

In DSM-5, which does not include a diagnostic category for MADD, the specifier ‘with anxious distress’ has been added to depressive and bipolar disorders [22], and thus patients presenting with co-morbid, subsyndromal, equally important anxiety and depressive symptoms may be coded to be suffering from ‘Other specified depressive disorder with anxious distress.’ This suggests that the authors of DSM-5 may have perceived mixed symptoms of anxiety and depression as depressive disorder with co-morbid anxiety. A study focusing on the consequences of illness on distress as well as on social, occupational, and psychological functioning showed, however, that the symptom profiles of patients with MADD were easily distinguishable from those of patients with MDD but were similar to those of patients with GAD from which they differed significantly only in more severe depressed mood and less severe somatic symptoms of anxiety [82]. The authors concluded that in ICD-10 the placement of MADD in one group with other anxiety disorders (F41) is therefore appropriate.

## Diagnosis determines treatment

Although anxiety and depression symptoms in MADD are subsyndromal by definition, they may nevertheless cause similar levels of distress and disability as those observed in patients with a syndromal diagnosis [e.g., 7, 10, 18, 83, 84] and bear the risk of exacerbation to a syndromal disorder [e.g., 85–88] and thus warrant clinical recognition and require appropriate treatment [89]. Effective treatment of MADD is not just an end in itself, but a major preventive intervention that could potentially alter the future course of a patient’s psychiatric illness [90].

Epidemiological data and reports from clinical practice suggest, however, that patients suffering from subsyndromal anxiety or depressive symptoms are often underrecognized and, consequently, undertreated [64, 91, 92]. We would therefore like to emphasize the importance of an appropriate diagnostic category in order to guide appropriate treatment. One reason for the underrecognition of MADD may be the lack of a suitable diagnostic category in the ‘DSM world’ and the vagueness of, or difficulty in using, the diagnostic category provided in ICD-10 [23–25].

Particularly in primary care, patients suffering from anxiety and/or depression are likely to present with somatic complaints (e.g., muscle tension, headache, palpitations, tachycardia, shortness of breath, sexual dysfunction) rather than mental health symptoms [4, 78, 93] which may ‘mask’ an underlying affective disorder, including MADD. For example, in a study published by Kunik et al. [94], 26 % of the participants with chronic obstructive pulmonary disease also met the diagnostic criteria for both anxiety and depression, while another 35 % met the criteria for either anxiety or depression alone, but less than 40 % of those with anxiety and/or depression were treated for this disorder. This may be another important factor contributing to the underrecognition of MADD in primary care.

Since MADD is associated with significant disability and impaired health-related quality of life, but in most cases is not a life-threatening condition, treatment should focus on the restoration of daily living skills and social functioning as well as on the prevention of an exacerbation to a potentially more serious psychiatric disorder, and should include only very limited risk. Of note, the use of drugs whose bothersome and partly disabling adverse effects, such as anticholinergic reactions, headache, sedation, gastrointestinal complaints, somnolence, weight gain, sexual dysfunction, or even anxiety and co-morbid insomnia, could aggravate the symptoms they were prescribed to treat, should be avoided. Co-morbid anxiety and depression have been shown to respond favorably to cognitive behavioral therapy [95, 96]. However, psychotherapy is often no viable option, due to lack of places on treatment programs. Therefore drugs that provide symptom alleviation at minimal risk are particularly important.

Up to now, there are only few randomized treatment studies for MADD. Since no drug has been licensed for the disorder, the affected patients have to be treated practically ‘off label.’ Therefore, guidelines cannot recommend evidence-based treatments for this common diagnostic entity. If MADD will remain in ICD-11, this might encourage researchers to do controlled trials in this disorder. At the moment, the US Food and Drug Administration (FDA) and the European Medicines Agency (EMA) require a full set of licensing studies for every single anxiety disorder, meaning that a drug licensed for panic disorder cannot automatically be used for GAD, etc. This is in contrast to the substantial diagnostic overlap among all anxiety disorders. Moreover, there is no evidence for a differential indication of certain treatments for the different anxiety disorders. Instead, it has been shown that the effect sizes obtained with medications for the different anxiety disorders are very similar [97]. If, in the future, FDA and EMA could be convinced that a new treatment could simply obtain a marketing authorization for ‘all anxiety disorders,’ including MADD, instead of separately for every disorder, this would result in less expenditure of time, money, and patients exposed to placebo, thus making it easier and more ethical to relieve anxiety symptoms in the affected patients.

## Why MADD is an important diagnostic category in its own right

In addition to nosologically and scientifically motivated demurs about the validity and usefulness as a diagnostic category, there are also concerns that an inclusion of MADD into classification systems could lead to a ‘medicalization’ of ‘minor, self-limiting symptoms of distress’ [74] and to stigmatization and undermining of coping strategies [17, 80]. However, by making a case for a diagnostic category of MADD, we are not advocating to lower the bar for a diagnosis and thus to unnecessarily tag millions of moderately ‘neurotic’ individuals with a psychiatric label. Our concern is with patients who suffer profoundly from distress and disability and whose symptoms, when considered in their entirety, clearly possess pathological significance. Denying such patients an appropriate diagnosis could well imply to withhold the required treatment as well.

Studies prospectively investigating longitudinal data widely agree that approximately half of the patients diagnosed with MADD remit within about 1 year [27, 72, 74], only slightly more than identified for MDD or GAD [27]. Among the non-remitting patients a substantial proportion was found to progress to a syndromal depressive or anxiety disorder or to another syndromal psychiatric diagnosis [27, 72]. While it has been argued that subthreshold symptoms of anxiety and depression may thus mainly be either self-limiting or a prodromal stage to a syndromal condition, not warranting a diagnostic entity in its own right [27, 79], the same findings can also be interpreted as evidence that the development of a more severe form of affective disorder could likely be prevented in a substantial fraction of patients by early recognition and appropriate treatment of MADD, saving both patients’ suffering as well as healthcare and economic resources. Lamentably, factors allowing to reliably predict the course of MADD, and to differentiate between patients who are likely to remit spontaneously and those who are at a substantial risk of progression to a syndromal affective disorder, are yet to be identified [74].

Since individuals with subthreshold affective disorders may experience similar levels of distress and disability as patients with a syndromal diagnosis, patients with MADD also exhibit similar social and occupational dysfunction [90]. As shown for minor depression [98], subthreshold disorders like MADD are associated with similar indirect, non-medical costs (e.g., through production loss due to illness) as syndromal disorders. The recognition of MADD as a condition requiring treatment may thus result in increased costs for healthcare utilization, but may nevertheless be cost-effective from a societal perspective.

In day-to-day clinical practice, particularly in primary care, physicians diagnose and treat large numbers of patients who present with comparatively trivial, self-limiting disorders. A good example is the common cold which, although it usually subsides within 2 weeks untreated, may cause profound, subjective suffering and has an enormous economic impact, mainly through loss of productivity [99, 100]. Of course, treatment of the common cold appears to be perfectly justified both from a clinical and from an economic perspective, since (a) patients suffer, (b) there is a certain risk of much more severe and difficult to treat complications and exacerbations, and (c) secondary costs, resulting from disability, may be reduced by an acceleration of recovery.

We have never heard of any criticism of common cold treatment founded in the conviction that a diagnosis of the disorder could lead to unjustified medicalization and stigmatization of millions of individuals who suffer from minor, self-limiting symptoms. This is because somatic disorders still appear to be perceived as something more ‘acceptable’ and less stigmatizing than psychiatric disorders, both in the general population and in the medical community. One thing that somatic and psychiatric disorders have in common is that patients suffer. Since they do, physicians should attempt to assign a matching diagnosis and to initiate appropriate treatment. Rather than to lament the potential stigmatization through mental illness, and denying an appropriate diagnosis, the medical community should better work toward an end of the stigmatization of mental illness in general.

The inclusion of the diagnostic category of MADD into our classification systems will help patients to gain access to appropriate treatment early. At least, successful treatment will reduce their suffering, even if the symptoms would otherwise have subsided after some time untreated. Moreover, there is also a fair chance that early treatment will prevent an exacerbation to a more serious condition. This is why we think that a diagnostic category of MADD is both justified and helpful in clinical practice.
